# A Monstrosity of Sex—The Case of John G. Allen

**Published:** 1869-05

**Authors:** L. E. Nagle


### A Monstrosity of Sex—The case of John G. Allen.

BY L. E. NAGLE, M. D.

Late in the year 1866, the person—John G. Allen—was arrested by the police, in the city of New Orleans, on a charge of being a female. She was masquerading in male apparel, and after a casual examination, was ordered to dress as a female, and was then discharged from custody. In this garb I found the person acting in the capacity of a servant, and answering to the name of Katie. From the person I learned that the parents had decided at his birth that he was a boy, and had treated him as such; and that he had always been recognized as a boy.

This person was nineteen years old in January, 1867. The general appearance is very feminine. The upper lip, chin and cheeks do not present any hirsute evidences. The face, hair and general features are those of a scrofulous female. The neck and shoulders are delicate, and have outlines of conformation similar to the appearance of a woman. The breasts are like those of a female child of twelve years of age. The nipples are very small, and have very little areola. The outlines of the abdomen and the swell of the hips, thighs and beautifully modeled legs, tapering delicately and ending with small, well-formed feet, all indicate female peculiarities. The shrinking sensitiveness, manners and voice are also very feminine. These, however, are the same that would be seen in a person who had been castrated in childhood.

I discovered in this person but one organ of sexuality. This is a penis, which is about an inch and a half long and a half inch in diameter. There are a miniature glans penis, faintly outlined, and an ordinary sized meatus, occupying their correct anatomical positions. The prepuce consists of numerous small, corrugated folds, and constantly covers the glands, thus presenting an entirely distinct form from a clitoris. A urethra passes from the meatus in nearly the proper course as found in males, and the orifice and sphincter are controlled in the manner usual to males. The base of the penis is in the natural situation. There is not the slightest trace of scrotum, testicles, or prostrate gland. There are no evidences of an attempt at labia. The perineum is rather full, but the *raphe* does not exhibit any appearance that nature ever attempted to establish a vaginal aperture. A male catheter was introduced through the urethra with much ease. The finger was introduced into the rectum, and the exploration assisted by the point of the catheter did not procure any evidence of a trace of a vaginal canal. The examination discovered to me rather a less quantity of interstitial tissue than is usually found in either sex. The exploration did not discover the slightest evidence of folds of vagina or a uterus.

The pulse is strong, and the hand more delicately formed than we usually find in males. The mons-veneris is covered with a small quantity of hair, which is of a very fine texture, and completely surrounds the base of the penis. The general admeasurement of the pelvis is less than the average in females, though the general appearance of the pelvic formation is very feminine, in all its delicate outlines, contour and structure.

This person states that he sometimes has undefinable copulative desires, which doubtless arise from a mental excitation. He thinks they are rather stronger in inclination for communication with males. During a long examination, in which much manipulation and friction necessarily occurred, we did not detect any signs of passion or sexual excitement in the person. He also informed us that he seeks the society and protection of men, and that his disposition is to associate with males, rather than with the opposite sex.

There has never been any secretion from the penis or any other organ of the body, which would indicate vicarious menstruation or seminal evacuation. The person is of hereditary scrofulous habit. Ulcers occasionally appear on the neck, shins, in the nose and other parts of the body. Epistaxis occurs occasionally, but is not periodical or traceable to any menstrual or unusual cause apart from sensitive membranous structure, consequent on this scrofulous taint.

From all these data, I conclude that this person is merely a natural eunuch. Nature forgot to add genitals to him, and he was thus emasculated by default.

In January, 1867, I took the person before the medical class of the University of Louisiana and New Orleans School of Medicine, where he was examined by Surgeon Warren Stone, Professor S. M.

Bemiss, Surgeon Campbell, and others; and the same particulars I have described were elicited and pronounced. We immediately certified to the foregoing condition of the case, and declared it to be our conviction that he was entitled to wear, and should be permitted to wear, the habiliment usual to males. In accordance with the particulars therein certified to, and after a farther personal examination held before Mayor Monroe, his honor gave John G. Allen a certificate to the effect that he was a male person, ordered him to wear the garments of his sex, and guaranteed protection against arrest and molestation on this account.—*N. O. Journal of Medicine.*

